# Coordinated bacterial and plant sulfur metabolism in *Enterobacter* sp. SA187–induced plant salt stress tolerance

**DOI:** 10.1073/pnas.2107417118

**Published:** 2021-11-11

**Authors:** Cristina Andrés-Barrao, Hanin Alzubaidy, Rewaa Jalal, Kiruthiga G. Mariappan, Axel de Zélicourt, Ameerah Bokhari, Olga Artyukh, Khairiah Alwutayd, Anamika Rawat, Kirti Shekhawat, Marília Almeida-Trapp, Maged M. Saad, Heribert Hirt

**Affiliations:** ^a^DARWIN21, Biological and Environmental Sciences and Engineering Division, King Abdullah University of Science and Technology, Thuwal 23955, Saudi Arabia;; ^b^Red Sea Research Center, King Abdullah University of Science and Technology, Thuwal 23955, Saudi Arabia;; ^c^Department of Biology, College of Sciences, University of Jeddah, Jeddah 21589, Saudi Arabia;; ^d^Université Paris-Saclay, CNRS, French National Research Institute for Agriculture, Food and Environment (INRAE), Université Evry, Institute of Plant Sciences Paris-Saclay, Orsay 91405, France;; ^e^Princess Nourah Bint Abdulrahman University, Riyadh 17452, Saudi Arabia;; ^f^Max F. Perutz Laboratories, University of Vienna A-1030 Vienna, Austria

**Keywords:** RNA-Seq, plant–microbe interaction, plant growth–promoting bacteria, salt stress, sulfur metabolism

## Abstract

*Enterobacter* sp. SA187 is a root endophytic bacterium that maintains growth and yield of plants under abiotic stress conditions. In this work, we compared the metabolic wirings of Arabidopsis and SA187 in the free-living and endophytic interaction states. The interaction of SA187 with Arabidopsis induced massive changes in bacterial gene expression for chemotaxis, flagellar biosynthesis, quorum sensing, and biofilm formation. Besides modification of the bacterial carbon and energy metabolism, various nutrient and metabolite transporters and the entire sulfur pathway were up-regulated. Under salt stress, Arabidopsis resembled plants under sulfate starvation but not when colonized by SA187, which reprogramed the sulfur regulon of Arabidopsis. In accordance, salt hypersensitivity of multiple Arabidopsis sulfur metabolism mutants was partially or completely rescued by SA187 as much as by the addition of sulfate, L-cysteine, or L-methionine. Many components of the sulfur metabolism that are localized in the chloroplast were partially rescued by SA187. Finally, salt-induced accumulation of reactive oxygen species as well as the hypersensitivity of LSU mutants were suppressed by SA187. LSUs encode a central regulator linking sulfur metabolism to chloroplast superoxide dismutase activity. The coordinated regulation of the sulfur metabolic pathways in both the beneficial microorganism and the host plant is required for salt stress tolerance in Arabidopsis and might be a common mechanism utilized by different beneficial microbes to mitigate the harmful effects of different abiotic stresses on plants.

## Significance

Although plant growth–promoting bacteria (PGPB) enhance the performance of plants, only a few mechanisms have been identified so far. We show that the sulfur metabolisms in both PGPB *Enterobacter* sp. SA187 and Arabidopsis plants play a key role in plant salt stress tolerance. Salt stress induces a sulfur starvation response in plants that is attenuated by SA187. Arabidopsis sulfur metabolic mutants are hypersensitive to salt stress but can be rescued by SA187. Most plant sulfur metabolism occurs in chloroplasts and is linked to stress-induced accumulation of reactive oxygen species that is suppressed by SA187. This work reveals that plant salt stress tolerance requires the coordinated regulation of the sulfur metabolic pathways in both beneficial microbe and host plant.

Plant growth–promoting bacteria (PGPB) have the capability to establish mutualistic associations with plants resulting in the increase of plant growth. PGPB can use different strategies to reduce the vulnerability of plants to biotic and abiotic environmental stresses (1). PGPB can live in the soil (rhizosphere) surrounding the plant roots, epiphytically attached to roots, stems, or leaves surfaces, or as endophytes inside plant tissues. PGPB are able to promote the growth of plants by different mechanisms such as nutrient uptake from soil (phosphate, nitrogen, iron, etc.), modulation of plant hormone levels (auxins, ethylene, abscisic acid, etc.), or enhancement of plant resistance to pathogens by the activation of defense mechanisms referred to as Induced Systemic Resistance (ISR) or the production of antimicrobials (2, 3). During the last decades, PGPB have gained interest as biotechnological tools with applications in agriculture as alternatives to traditional chemical fertilizers and pesticides (4).

Monitoring the modifications in metabolic pathways is a powerful approach to identify factors and regulatory processes involved in beneficial plant–microbe interaction (5). Upon interaction of PGPB with host plants, a global alteration of gene expression and metabolic pathways is triggered in both interacting organisms. RNA sequencing (RNA-Seq) has proved to provide highly accurate and sensitive transcriptional profiling of both microbes and host plants and has contributed to substantial gain in biological insight on both sides of the interaction (6–8). Nevertheless, the application of RNA-Seq in the context of plant–microbe interactions has focused on the host plant responses (9–12). A number of studies have investigated transcriptome changes in different PGPB species cultured under conditions intended to mimic an endophytic lifestyle (13–20). However, studies on the transcriptional changes in PGPB upon host interaction under *in planta* conditions remains limited (21).

During the last years, the potential of the endogenous microbiomes of desert plants has been investigated for their use as biofertilizers (22, 23). Strains isolated from desert plants have proven their efficiency in promoting growth of agronomic important crops such as canola, cucumbers, and alfalfa (24, 25). One of these strains, *Enterobacter* sp. SA187, is an endophytic PGPB that was isolated from root nodules of the endogenous desert plant *Indigofera argentea*, from the Jizan region in Saudi Arabia (26). SA187 has been described to promote multistress tolerance under desert farming conditions in the crop plant alfalfa and under laboratory conditions in the model plant Arabidopsis (12). Our recent report on Arabidopsis demonstrated that the promotion of salt stress tolerance by SA187 is mediated through activation of the plant ethylene signaling pathway. Furthermore, the results suggested that SA187 induces salt stress tolerance by producing 2-keto-4-methylthiobutyric acid (KMBA) (12), which is known to be converted into ethylene *in planta* (27). To expand our understanding on the molecular mechanisms underlying the beneficial association of SA187 with Arabidopsis, we here compare the transcriptomes of SA187 and Arabidopsis before and after establishing functional interaction under both normal and salt stress conditions. We show that the endophytic colonization of Arabidopsis by SA187 is accompanied by a genetic reprogramming of host and bacterial primary and secondary metabolic pathways, revealing the bacterial and plant sulfur metabolic pathways as key players in the beneficial plant–microbe interaction.

## Results

### Reprogramming of the SA187 Metabolic Pathways by Interaction with Arabidopsis.

#### SA187 transcriptome analysis upon colonization of Arabidopsis*.*

**Fig. 1.**
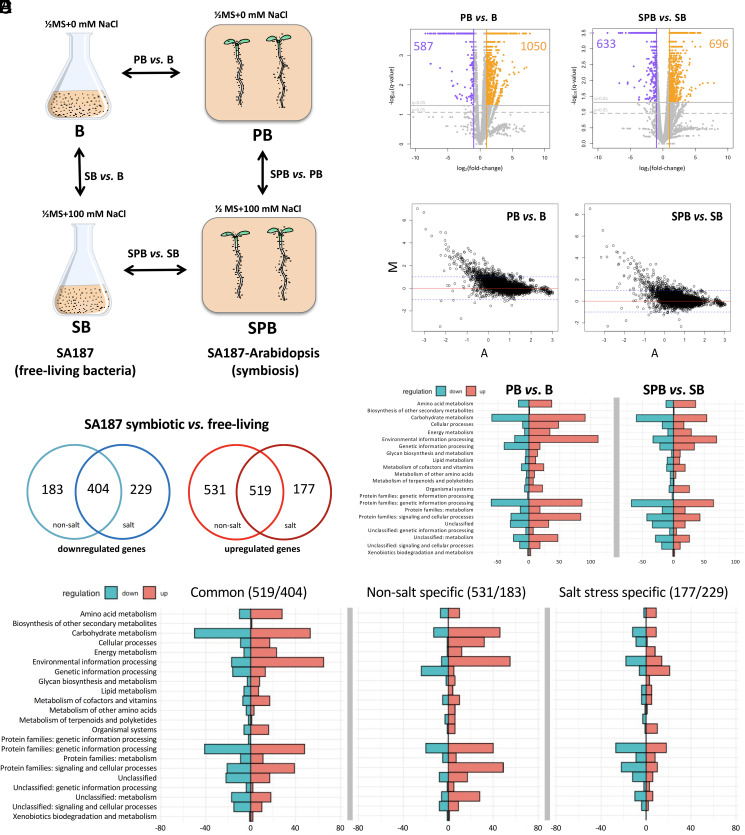
Scheme of experimental setup: SA187 was incubated in 1/2MS without (B) or with 100 mM NaCl (SB) for 4 h at 28 °C. Arabidopsis seedlings were germinated for 5 d, with or without SA187 before transfer to fresh 1/2MS plates for 12 d without (P, PB) or with 100 mM NaCl (SP, SPB) and vertically grown for 12 d (22 °C, 16/8 h light/dark cycle) (*A*). Volcano plots of SA187 DEGs in free-living and endophytic conditions with and without salt: to analyze the effect of salinity, SA187 in 1/2MS + 100 mM NaCl (salt stress) was compared to SA187 grown in 1/2MS (mock) in both free-living and plant-associated conditions (*B*). MA plots: representation of the gene expression fold change (M-axis) versus the mean gene expression values (A-axis) for each comparison. Dotted blue line marks the differential expression fold change = 2 [log2(fold change) = 1], those genes showing M > 1 as up-regulated and M < −1 as down-regulated. M = log2(log10(FPKM2)/(log10(FPKM1)), A = 1/2log2(log10(FPKM1)*log10(FPKM2)). Zero values of FPKM were ignored. Scatter plots were generated with R (Rstudio version 1.0.136) (*C*). Venn diagram showing up- and down-regulated genes that are common or specific for mock and salt stress conditions, comparing SA187 endophytic to free-living state (q-value <0.05) (*D*). KO functional analysis: metabolic processes regulated in response to the adaptation to the plant-associated lifestyle under nonsalt (PB versus B) and salt stress conditions (SPB versus SB) (*E*). Metabolic processes up- and down-regulated in response to plant associations that are common in both control and salt stress conditions (*Left*) or specific to control condition (*Center*) or salt stress (*Right*) (*F*). *y*-axis = KEGG functional categories. *x*-axis = number of genes or KO identifiers in KEGG knowledgebase from each subset of regulated genes in our analysis. Up-regulated metabolic processes are in red and down-regulated in blue.

To understand the processes that are induced by the endophytic interaction with Arabidopsis roots in SA187, RNA-Seq analysis was performed as shown in Fig. 1*A*. SA187 interaction with Arabidopsis roots was evaluated by electron microscopy (*SI Appendix*, Fig. S1*A*) and bacterial proliferation levels, which were similar under both nonsalt and salt conditions, were assessed by qRT-PCR (*SI Appendix*, Fig. S1*B*). Approximately, 16 to 20 million paired-end reads were obtained from the single bacterial and 60 to 82 million reads from dual SA187-Arabidopsis RNA-Seq (*SI Appendix*, Table S1). Due to the small bacteria/plant ratio in the RNA obtained from dual samples, the majority of sequencing reads mapped to the Arabidopsis TAIR10 (The Arabidopsis Information Resource) genome, with only <1% of bacterial reads (*SI Appendix*, Table S1). Despite the small number of bacterial reads obtained, the analysis of sequencing depth confirmed the quality of all RNA-Seq libraries (*SI Appendix*, Fig. S1*C*). Pearson’s linear regression coefficients, FPKM (fragment per kilobase per million mapped reads) profiles, and the distribution of mapped reads showed a good correlation among the biological replicates (*R*^2^ > 0.98 and ∼0.90 for free-living and endophytic samples, respectively) with the exception of replicate SB1 (*R*^2^ = 0.73), which was hence excluded from further analysis (*SI Appendix*, Fig. S1 *D* and *E*).

By applying a differential expression cutoff of q-value = 0.05 and fold change >2, RNA-Seq data revealed that the endophytic interaction of SA187 with Arabidopsis induced significant changes in the bacterial transcriptome irrespective of the presence or absence of salt. Under nonsalt conditions, endophytic SA187 showed 1,050 genes up-regulated and 587 down-regulated compared to free-living bacterium (PB versus B) (Table 1 and Fig. 1*B*). Under salt stress, the endophytic versus free-living comparison showed 696 up-regulated and 633 down-regulated genes (SPB versus SB) (Table 1 and Fig. 1 *B* and *C*). The addition of 100 mM NaCl to the growth medium only had a minor effect on the gene expression of free-living and endophytic SA187 (SB versus B, SPB versus PB) (Table 1 and *SI Appendix*, Fig. S2). According to these data, a core set of 519 up-regulated and 404 down-regulated genes in SA187 is involved in the endophytic lifestyle under both salt and nonsalt conditions (Fig. 1*D*), and similar biological processes are modified in all endophytic samples (Fig. 1 *E* and *F*). In total, 50 key genes involved in the endophytic lifestyle of SA187 were selected for qRT-PCR validation. The large majority of qRT-PCR expression patterns of SA187 genes confirmed the reliability of the RNA-Seq results (*SI Appendix*, Figs. S3 *A* and *B* and S4*A*).

**Table 1.** Normalized DEGs (FPKM, q-value < 0.05) in *Enterobacter* sp. SA187

Investigated effect	Salt stress (100 mM NaCl)				Interaction with Arabidopsis			
Comparison	SB versus B (free living)	%*	SPB versus PB (endophytic)	%*	PB versus B (0 mM NaCl)	%*	SPB versus SB (100 mM NaCl)	%*
Up-regulated	79	1.71	5	0.11	1050	22.8	696	15.1
Down-regulated	169	3.67	15	0.32	587	12.7	633	13.7
Total regulated	248	5.38	20	0.43	1,637	35.5	1,329	28.8

*Percentage of SA187 genome identified to be regulated.

#### Genes involved in motility, chemotaxis, quorum sensing, and biofilm formation are induced in SA187 upon interaction with Arabidopsis*.*

To obtain insight into the biological functions of the identified differentially expressed genes (DEGs), we used the Kyoto Encyclopedia of Genes and Genomes to perform KEGG Orthologous (KO) and Brite mapper analyses (*SI Appendix*, Fig. S5).

Biological processes involved in the colonization of the host plant, such as chemotaxis, flagellar assembly, quorum sensing (QS), and biofilm formation were up-regulated during the endophytic interaction of SA187 with Arabidopsis (*SI Appendix*, Figs. S5 and S6 and Dataset 4). Interestingly, the genome of SA187 contains five paralogous genes coding for flagellin (FliC) (26). While three out of the five genes were poorly expressed in all conditions, two flagellin genes showing the highest homology (77.3% identical) to *Pseudomonas aeruginosa flg22* motif (28), SA187PBcda_000004844 (fliC.4) and SA187PBcda_000004845 (fliC.5), were induced six- to eightfold upon plant interaction (*SI Appendix*, Figs. S3*A* and S4*A* and Dataset 4). Moreover, a number of methyl-accepting chemotaxis proteins and the two-component system (TCS) CheA/CheB, involved in the signaling cascade controlling flagellar assembly, together with chemotaxis proteins CheW and CheZ, were also up-regulated upon plant interaction (*SI Appendix*, Fig. S3*A* and Datasets 4 and 5).

In the endophytic state, TCS of SA187 involved in QS, QseC/QseB and FusK/FusR, were up-regulated (Datasets 3 and 5). The AI-2 transporter (LsrBCDA), together with the *lsr* operon transcriptional repressor LsrR and terminator LsrF, were also up-regulated (Datasets 1 and 3). Additionally, a number of genes involved in biofilm formation, including subunits of the curli fibers biosynthetic complex were induced: CsgB and CsgC, as well as poly-N-acetyl-glucosamine (PGA) synthase subunits PgaA and PgaC (Dataset 6).

#### Nutrient and metabolite transport, as well as carbon metabolism and oxidative phosphorylation, are induced in SA187 during endophytic interaction with Arabidopsis*.*

In the SA187 endophytic state, the phosphotransferase systems (PTS) involved in the import of glucose, sucrose, 2-O-α-mannosyl-D-glycerate, cellobiose, and β-glucoside were strongly up-regulated (*SI Appendix*, Fig. S6 and Dataset 2). Many ABC transporters were also up-regulated, including the transporters of sulfate CysPUWA, maltose/maltodextrine MalEFGK, D-methionine MetNIQ, and the signaling molecule autoinducer 2 (AI-2) LsrABCD (*SI Appendix*, Fig. S6 and Dataset 1). Interestingly, subunits involved in the transport of osmoprotectants, such as glycine-betaine, proline, spermidine, and putrescine, as well as ABC.SP.A, ABC.SP.P1, and ABC.SP.S were found to be down-regulated (Datasets 1 and 3).

A large number of bacterial genes of the carbohydrate metabolism were up-regulated during the beneficial interaction. While glycolysis/gluconeogenesis and the pentose phosphate pathway were down-regulated, the tricarboxylic acid (TCA) cycle and the glyoxylate pathways were up-regulated (*SI Appendix*, Fig. S5 and Datasets 10 and 11). Among the down-regulated genes, we identified fructose phosphokinase (PfkB) (Dataset 11), one of the key regulatory points that make glycolysis irreversible, and pyruvate dehydrogenase (PoxB), involved in another rate-limiting step of glycolysis (Dataset 11). On other hand, citrate synthase was up-regulated (Dataset 11), suggesting that SA187 makes preferential use of the TCA cycle to produce energy in the form of GTP and NADH *in planta*.

Oxidative phosphorylation, the metabolic process that generates the energy needed for almost all vital processes, was up-regulated during the interaction, including *O*-cytochrome (CyoCDE) and several subunits of the F_0_/F_1_-ATP synthase, which convert the transmembrane proton gradient into ATP (Dataset 12). Similarly, several subunits of NADH-dehydrogenase (complex I) and succinate dehydrogenase (SDH) (complex II), which are also part of the TCA cycle, were also strongly up-regulated during beneficial interaction (*SI Appendix*, Fig. S6 and Datasets 11 and 12).

#### Modification of the SA187 primary and secondary metabolic networks by Arabidopsis*.*

Sugar availability was suggested to be a main determinant of the microbial metabolic adjustment to an endophytic state (16). Upon interaction with Arabidopsis, the strong up-regulation of the SA187 ABC transporters and PTS suggests the active uptake of multiple nutrients from the plant host. These results agree with reports from *Bacillus amyloliquefaciens* FZB42, SQR9, or *Enterobacter* sp. 638 (13, 15, 16), showing that glucose, maltose, or inositol in maize root exudates induced the expression of the respective bacterial ABC and PTS transport systems. To assess if the endophytic SA187 gene expression profile is mainly driven by sugar availability from the host plant, we analyzed the expression of 40 endophytic SA187 marker genes during growth in media supplemented with 1% sucrose. Of the marker genes, 43% showed an expression profile similar to an endophytic state (*SI Appendix*, Figs. S3 *C–E* and S7), indicating that nutrient availability is a major driver in the regulation of the SA187 metabolism. Nonetheless, 57% of bacterial genes did not respond to the addition of sucrose to the growth medium (*SI Appendix*, Fig. S7), suggesting that additional factors are also responsible for modifying the SA187 metabolic network during Arabidopsis interaction.

#### Interaction with Arabidopsis modifies the bacterial sulfur metabolism in SA187.

**Fig. 2.**
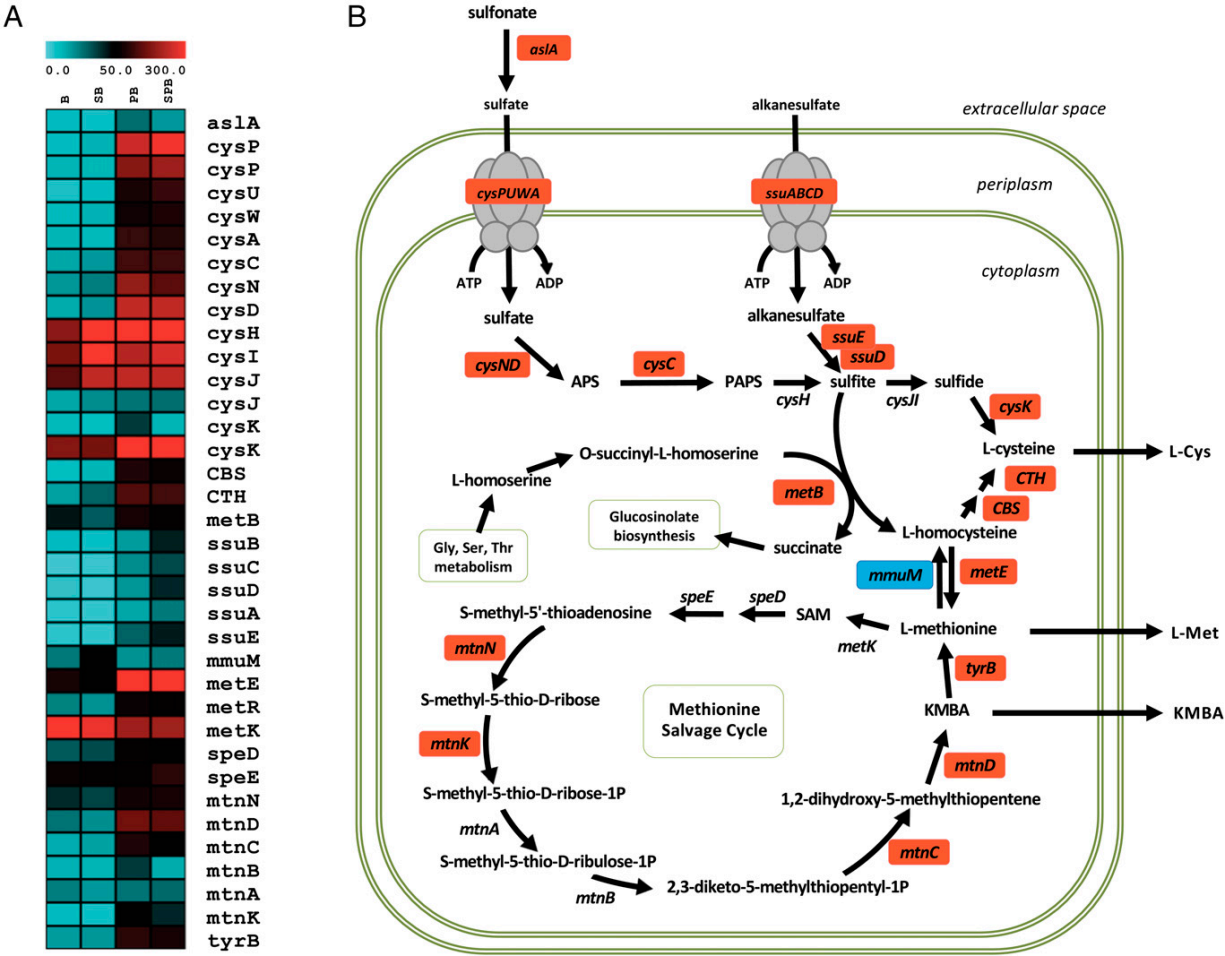
Heatmap constructed using up-regulated (red) and down-regulated (blue) SA187 genes encoding sulfur metabolic transporters and enzymes during endophyte colonization of Arabidopsis compared to free-living bacteria (*A*). Up-regulation of the SA187 sulfur metabolic pathway by Arabidopsis. APS = adenosine 5′-phosphosulfate, PAPS = 3′-phosphoadenosine-5′-phosphosulfate, KMBA = 2-keto-4-methylthiobutyric acid (*B*).

Our previous work identified the methionine salvage pathway intermediate KMBA as an important metabolite that is provided by SA187 to induce salt tolerance in Arabidopsis (12). Moreover, it was shown that an active metabolism of SA187 is needed as heat-inactivated bacteria had also lost its beneficial effects (12). However, it was unclear from where the sulfur for KMBA formation originated and whether only the methionine salvage pathway was up-regulated. Our comparative transcriptome analysis clearly showed that, upon interaction with Arabidopsis, the SA187 *aslA* gene, mobilizing the sulfur of the soil, was up-regulated in parallel with the ABC sulfate transporter CysPUWA (Fig. 2 and *SI Appendix*, Fig. S6). Upon cellular uptake into SA187, sulfate adenylyltransferase (CysND) converts sulfate into APS (adenosine 5′-phosphosulfate) followed by adenylyl kinase (CysC) to produce PAPS (3′-phosphoadenosine-5′-phosphosulfate), which can then be converted into sulfite (Fig. 2 and *SI Appendix*, Fig. S6). Up-regulation of the SsuABC transporter expands the possibilities of SA187 to use alkane sulfates from the environment. Consistently, FMNH_2_-dependent sulfonate monooxygenase (SsuD) and the FMN-reducing enzyme that provides SsuD with FMNH2 (SsuE), which convert alkane sulfonates into sulfite, were also up-regulated (Fig. 2 and *SI Appendix*, Fig. S6 and Datasets 1, 9, and 10). Upon enzymatic reduction of sulfite into sulfide, cysteine kinase (CysK) catalyzes the synthesis of L-cysteine. The enzymes involved in the conversion of L-cysteine to L-methionine, CBS, TCH, and MetE, together with the MetE transcriptional regulator MetR, were also highly up-regulated upon SA187 interaction with Arabidopsis (Fig. 2 and *SI Appendix*, Fig. S6 and Datasets 9 and 10). In addition, the genes encoding the enzymes of the methionine salvage pathway TyrB, MtnN, MtnK, MtnC, MtnD, and SpeD showed significant up-regulation during plant interaction (Fig. 2 and *SI Appendix*, Fig. S6 and Datasets 9 and 10). These results show that the entire sulfur metabolism and not only the methionine salvage pathway of SA187 is strongly induced upon interaction with Arabidopsis.

### Modification of the Arabidopsis Metabolism upon Endophytic SA187 Interaction.

#### SA187 enhances sulfur levels in Arabidopsis roots.

**Fig. 3.**
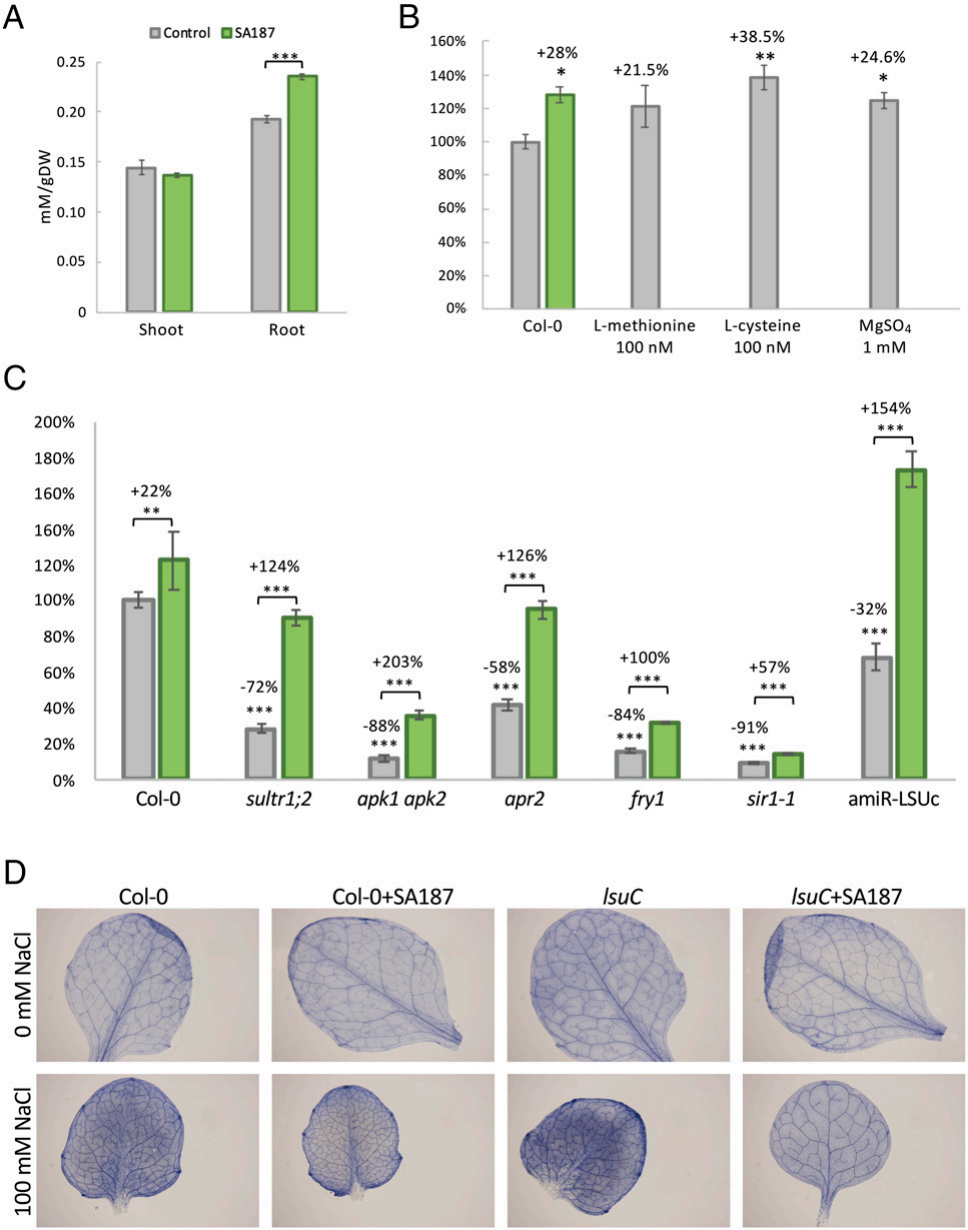
Effect of SA187 inoculation in Arabidopsis on 1/2MS + 100 mM NaCl. Shoot and root sulfur levels of Arabidopsis grown under salt stress, in the presence or absence of SA187. Values represent means of three biological experiments, each in three technical replicates (*n* = 9). Error bars represent SE. Asterisks indicate the statistically significant difference based on the Mann–Whitney *U* test (***P < 0.001) when compared to mock plants (*A*). Total fresh weight beneficial index of 21-d-old plants treated with SA187, 100 nM L-methionine, 100 nM L-cysteine, or 1 mM MgSO_4_, on 100 mM NaCl. Values represent means of 3 biological experiments, each in 10 technical replicates (*n* = 30). Error bars represent SE. Asterisks indicate the statistically significant difference based on the Mann–Whitney *U* test (*P < 0.05; **P < 0.01) when compared to mock plants (*B*). Total fresh weight beneficial index of noncolonized (gray) and SA187-colonized (green) 21-d-old wild-type, *sultr1*, *apk1/2*, *apr2*, *fry1*, *sir-1*, or amiR-LSUc plants, on 1/2MS + 100 mM NaCl. Values represent means of 3 biological experiments, each in 12 technical replicates (*n* = 36). Error bars represent SE. Asterisks indicate the statistically significant difference based on the Mann–Whitney *U* test (**P < 0.01; ***P < 0.001) when compared to noninoculated wild-type plants (*C*). Accumulation of superoxide radicals (visualized by nitroblue tetrazolium staining) in 0 (control) and 100 mM NaCl (salt stress), in wild-type and amiR-LSUc mutant plants (*D*).

We next performed Inductively Coupled Plasma–Optical Emission Spectroscopy (ICP-OES) analysis of Arabidopsis shoots and roots that had been cultivated under both nonsalt and 100 mM NaCl conditions for 21 d in the absence or presence of SA187. Interestingly, under salt stress, we found significantly higher sulfur levels in SA187 colonized Arabidopsis roots but not in shoots (Fig. 3*A* and *SI Appendix*, Fig. S8*A*). Salt stress strongly influences plant growth and photosynthesis, possibly due to the number of iron-sulfur cluster proteins of the photosynthetic apparatus of chloroplasts, disturbances of which result in aberrant electron transport and reactive oxygen species (ROS) generation (29). The sulfur metabolism has also been connected to the establishment of a successful plant–microbe interaction of both beneficial and pathogenic microbes (30, 31).

#### Sulfate positively affects salt stress tolerance of Arabidopsis*.*

Increased sulfate concentrations in the medium have also been reported to help plants to withstand salt stress conditions (32). Therefore, we compared the growth of endophytic SA187 plants under salt stress conditions (control, 1/2 MS + 100 mM NaCl, 0 mM MgSO_4_) with the addition of MgSO_4_ into the medium (treated, 1/2 MS + 100 mM NaCl, 1 mM MgSO_4_). The addition of sulfate showed similar significant beneficial effects on plants as colonization with SA187 (Fig. 3*B* and *SI Appendix*, Fig. S8*B*). Since our dual transcriptome analysis indicated that not only sulfate uptake but also cysteine and methionine synthesis are up-regulated in SA187 (12), we determined internal cystine (product of the oxidation of 2 molecules of cysteine) and methionine levels in SA187-colonized Arabidopsis. In contrast to shoots, both cystine and methionine levels were significantly higher in SA187-colonized roots, especially under salt stress conditions (*SI Appendix*, Fig. S9). We also tested whether 100 nM L-cysteine or L-methionine could have a beneficial effect on plant growth under salt conditions. L-methionine, L-cysteine, and MgSO_4_ showed similar beneficial effects, in the same range as when plants were colonized by SA187 (Fig. 3*B*). A beneficial effect of these compounds was also observed on plants grown under nonsalt conditions (*SI Appendix*, Fig. S8*B*).

#### SA187 modulates sulfur metabolism–related gene expression in Arabidopsis*.*

**Fig. 4.**
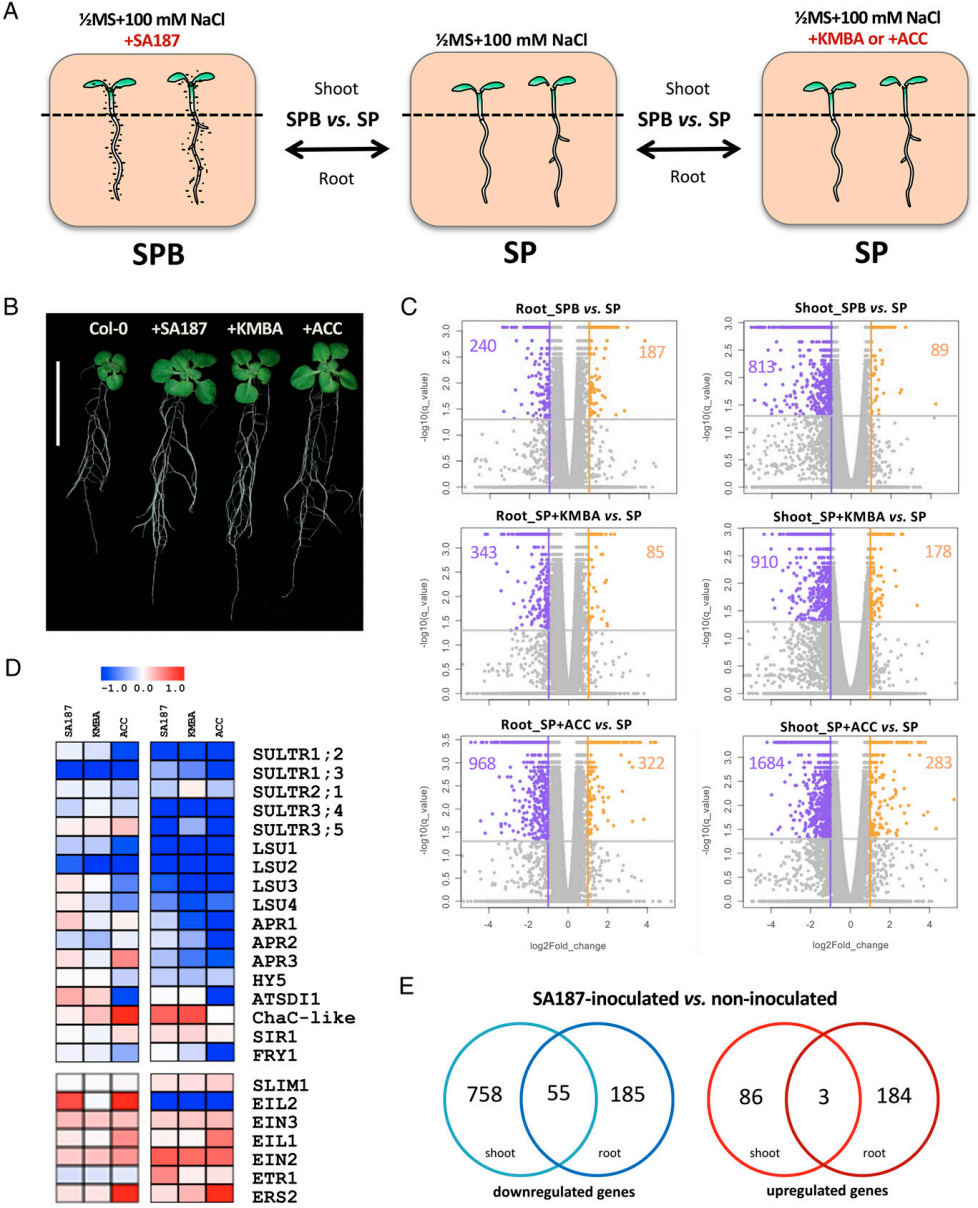
Effect of SA187 inoculation in Arabidopsis shoots and roots on 1/2MS + 100 mM NaCl, experimental setup (*A*). Phenotypic assessment of the beneficial effect on the growth of 21-d-old Arabidopsis seedlings of the three treatments, on 1/2MS + 100 mM NaCl: inoculation with SA187 and addition of 100 nM KMBA or 100 nM ACC to the growth medium. Col-0 = untreated Arabidopsis as control (*B*). Volcano plots of Arabidopsis roots and shoots DEGs, on 1/2MS + 100 mM NaCl, for each of the three treatments: +SA187 (SPB versus SP), +100 nM KMBA (SP + KMBA versus SP), or +100 nM ACC (SP + ACC versus SP) (*C*). Heatmap constructed using sulfur and ethylene signaling Arabidopsis genes that are statistically significantly up-regulated (red) and down-regulated (blue) by each of the three treatments: +SA187, +100 nM KMBA, +100 nM ACC (*D*). Venn diagram showing up- and down-regulated genes that are common or specific for Arabidopsis shoots and roots, comparing SA187 endophytic to free-living state (q-value <0.05) (*E*).

To get a deeper understanding of the changes in Arabidopsis plants induced by the root endophyte SA187, we generated a root and shoot transcriptome of 21-d-old plants that were grown on 100 mM NaCl either in the absence or presence of SA187, or treated with 100 nM KMBA, compared with plants treated with 100 nM ACC as positive control (Table 2 and Fig. 4 *A–C*). We obtained a total of ∼40 to 64 million paired-end reads. Pearson’s linear regression coefficients, FPKM profiles, and the distribution of mapped reads showed a good correlation among the biological replicates: 0.98 ≥ *R*^2^ > 0.87. By applying a differential expression cutoff of q-value = 0.05 and fold change >2, RNA-Seq data showed that SA187 colonization induced the down-regulation of 813 and up-regulation of 89 genes in shoots, whereas 240 down- and 187 up-regulated genes were identified in SA187-colonized roots (Table 2). The SA187-derived active compound KMBA induced the down-regulation of 910 and up-regulation of 178 genes in shoots, whereas 343 down- and 85 up-regulated genes were identified in KMBA-treated roots (Table 2). The ethylene precursor ACC induced the down-regulation of 1,684 and the up-regulation of 283 genes in shoots, whereas 968 down- and 322 up-regulated genes were identified in ACC-treated roots (Table 2 and Fig. 4*C*). Notably, the DEGs were specific to the shoot and/or root tissues, with a low number of common DEGs between plant tissues (Fig. 4*E*). This observation supports the hypothesis of the tissue-specific effect of the beneficial microbe SA187 despite the overall beneficial growth under salt stress.

**Table 2.** Normalized DEGs (FPKM, q-value < 0.05) in *Arabidopsis thaliana* Col-0

Investigated treatment	SA187 inoculation		KMBA 100 mM		ACC 100 mM	
Comparison	SPB versus SP	%*	SP_ACC_ versus SP	%*	SP_KMBA_ versus SP	%*
	Root					
Up-regulated	187	0.68	85	0.31	322	1.18
Down-regulated	240	0.88	343	1.25	968	3.54
Total regulated	427	1.56	428	1.56	1,290	4.71
	Shoot					
Up-regulated	89	0.33	178	0.65	283	1.03
Down-regulated	813	2.97	910	3.32	1,684	6.15
Total regulated	902	3.30	1,088	3.98	1,967	7.19

*Percentage of *Arabidopsis* genome identified to be regulated.

A Gene Ontology (GO) term enrichment analysis showed that the up-regulated gene set in shoots is enriched for proteins with functions in jasmonic acid metabolism, whereas those of roots showed enrichment in root morphogenesis (*SI Appendix*, Fig. S8). Interestingly, the down-regulated genes in both shoots and roots showed significant enrichment for terms of defense responses and salicylic acid processes (*SI Appendix*, Fig. S8). A more refined gene by gene analysis revealed a significant regulation of sulfur metabolic genes in roots and shoots (Fig. 4*D*). In shoots and roots, transcript levels of sulfate transporters (*SULTR*), ATP sulfurylases (*ATPS*), APS kinases (*APK*), APS reductases (*APR*), sulfate reductase *SIR1*, and several sulfur-regulated transcription factors such as *SLIM1*, *EIL2*, *EIN3*, and *EIL1* were differentially regulated when compared to noncolonized plants (Fig. 4*D* and *SI Appendix*, S4*B*). Interestingly, many of these genes are part of the sulfur regulon, which not only regulate sulfur metabolism but are key factors the glucosinolate biosynthesis and ethylene signaling pathways (33).

#### Role of sulfur assimilation in SA187-induced salt stress tolerance in Arabidopsis*.*

We found that SA187 increases sulfur levels in Arabidopsis salt-stressed roots and enhances gene expression of a number of members of the sulfur regulon, indicating that SA187 might directly affect the sulfur uptake or metabolism of Arabidopsis (Fig. 4*D*). To test a role of the sulfate assimilation pathway in the beneficial SA187 interaction with Arabidopsis, we analyzed a number of sulfur metabolism mutants of Arabidopsis. As shown in Fig. 3*C*, *sultr1;2* (34), *apk1 apk2* (35), *apr2* (36), *sir1* (37), and *fry1* (38) mutants are hypersensitive to salt stress but could be significantly rescued by colonization with SA187. It should be noted that all mutants were already affected to some degree in growth under normal growth conditions (*SI Appendix*, Fig. S8*D*), but SA187 only exerted substantial rescue of the strong growth inhibitory phenotypes under salt stress (Fig. 3*C*). These results indicate that SA187 and the sulfur metabolism are tightly linked for conferring salt stress tolerance in Arabidopsis.

The majority of sulfur assimilation enzymes are localized in the chloroplast, suggesting that an intact chloroplast physiology is needed for proper functioning. In this respect, we noted that SA187-colonized Arabidopsis continue to grow under salt stress and show no down-regulation of photosynthesis-related genes, as shown for the genes encoding electron transport carriers in photosystem I and II, such as low quantum yield of photosystem ii 1 (LQY1), xyloglucan endotransglucosylase/hydrolase 6 (XTH6), acetyl-CoA:(z)-3-hexen-1-ol acetyltransferase (CHAT), deficient in cutin ferulate (DCF), and haloacid dehalogenase-like hydrolase (HAD) superfamily protein (*SI Appendix*, Fig. S4*B*).

#### SA187 suppresses salt stress–induced ROS accumulation in Arabidopsis*.*

Salt stress induces the generation of ROS in chloroplasts, and one of the most dramatic effects in salt grown plants is the down-regulation of the entire photosynthetic machinery (29). To test the possibility that SA187 protects Arabidopsis from excessive ROS levels, we monitored O_2_^–^ levels in SA187-colonized plants grown at 0 or 100 mM NaCl. As seen by nitroblue tetrazolium staining, SA187 did not change superoxide radical levels under nonstress conditions (Fig. 3*D*). However, previous results from our group show that the salt stress–induced increase in ROS levels in noncolonized Arabidopsis plants was significantly reduced upon SA187 colonization (Fig. 3*D*) (12). Since the sulfur metabolism is tightly linked to ROS protection via the synthesis of glutathione, one possible explanation for these results could be that SA187-colonized plants have higher redox capacity due to an improved GSH/GSSG ratio. Therefore, we determined GSH and GSSG levels in shoots and roots of SA187-colonized and noncolonized plants, in the absence and presence of salt stress (*SI Appendix*, Fig. S10). As expected, GSH/GSSG levels were strongly reduced in plants upon salt stress (*SI Appendix*, Fig. S10 *B* and *E*). Compared to noncolonized plants, shoots and roots of SA187-colonized plants showed higher GSH/GSSG ratios under nonstress conditions, but only roots of SA187-colonized plants showed strongly enhanced GSH/GSSG ratios under salt stress (*SI Appendix*, Fig. S10 *B* and *E*). In summary, these results support the notion that the SA187-induced Arabidopsis sulfur metabolism enhances glutathione levels to reduce damage by ROS.

#### SA187 suppresses the hypersensitive phenotype of lsu mutants.

Recently, a role of members of the *LOW SULFUR UP-REGULATED* (LSU) gene family (*LSU1-LSU4*) was reported in protecting the photosynthetic machinery under salt stress conditions (39). The LSU family was shown to directly interact and activate the chloroplast superoxide dismutase FSD2 and therefore functions in regulating plastid superoxide radical levels (39). To test a potential interaction of SA187 with the LSU network in Arabidopsis, we tested the amiR-LSUc line, expressing artificial microRNAs (amiRNAs) targeting all four members of the LSU gene family in Arabidopsis (39). As expected, *lsuC* lines showed much stronger accumulation of ROS under salt stress conditions (Fig. 3*D*), confirming its role in ROS protection. Interestingly, ROS accumulation of salt-stressed *lsuC* plants was completely suppressed upon SA187 colonization (Fig. 3*D*). This result is supported by the phenotypic analysis, showing that the growth reduction in amiR-LSUc plants is strongly complemented upon SA187 colonization (Fig. 3*C*), indicating that SA187 suppresses Arabidopsis salt stress–induced ROS accumulation in plastids via the LSU pathway.

## Discussion

Many PGPB have been described to interact with and exert a beneficial effect on plant growth and stress tolerance, but understanding the underlying molecular mechanisms has been hampered by the intrinsic complexity of the heterogeneous natural systems. Moreover, different bacterial strains and species use different strategies to help growth of plants under various conditions, and the interacting mechanisms are not only determined by the interacting microbes but also by the plant species, ecotype, developmental and physiological status, and the abiotic or biotic environment (4). Additionally, a switch of controlled growth to anarchic proliferation can result in the transformation from a beneficial to a pathogenic bacterial lifestyle (40). *Enterobacter* sp. SA187 provides salt tolerance to the crop alfalfa in open field trials and also to Arabidopsis under controlled laboratory conditions and can serve as a good model system to understand how beneficial microbes induce stress tolerance to plants. Compared to the small (5 to 10%) beneficial effect under nonsalt conditions, SA187 enhances plant growth to about 40 to 50% under salt conditions (12). Moreover, the bacterial metabolite KMBA, which was suggested to be transformed into ethylene *in planta*, promotes plant growth under salt stress. Our present analysis identifies the bacterial and plant sulfur metabolic pathways to be of primary importance in the beneficial interaction of SA187 with Arabidopsis. Our work unravels the importance of the coordinated regulation of the bacterial and plant sulfur metabolisms in plant stress tolerance. Moreover, our data provide a mechanistic understanding how sulfur metabolism and chloroplast functioning are linked with ethylene signaling in the context of salt stress in Arabidopsis.

The analysis of the SA187 metabolic processes during endophytic interaction with Arabidopsis revealed two large sets of genes. On one hand, we identified a set of genes that is mostly involved in the initial contact and the colonization of a host plant, such as chemotaxis, bacterial motility, QS and signaling, and biofilm formation. The other set of genes is related to the transport, exchange of nutrients as well as the carbon, sulfur, and energy metabolism.

### SA187 Colonization of the Plant Host.

The first steps in root endophyte colonization of plants consist of the chemotaxis and subsequent attachment of bacterial cells to the roots using cellulose-like fibers, colanic acid, and adhesion proteins (17). Chemotaxis and bacterial motility are often oppositely regulated to biofilm formation (15, 16, 18), which is controlled by QS, coordinating gene expression in a cell-density–dependent manner (41). In order to successfully colonize the plant roots, beneficial bacteria produce cellulose or cellulose-like fibers to form a biofilm (13, 15, 18). Upon stable colonization of Arabidopsis by SA187, a decrease in the expression of the bacterial genes for cellulose and curli fibers and the up-regulation of factors involved in biofilm formation were observed. The simultaneous up-regulation of bacterial genes involved in flagella biosynthesis and chemotaxis suggests that a heterogeneous population of SA187 exists even after the establishment of the endophytic state. While a fraction of bacterial cells might have become sedentary and form biofilms, another motile fraction might still spread through the constantly growing plant tissues.

### Modification of the Arabidopsis Stress Physiology by SA187.

In beneficial plant–microbe interactions, both partners benefit from the association showing extensive exchange of nutrients, metabolites, and information. However, so far little is known about how the expression of a large number of genes is coordinated in each partner. During salt stress of Arabidopsis plants, inhibition of growth and development is correlated with ROS accumulation and the down-regulation of photosynthesis and carbohydrate metabolism. Importantly, SA187 colonization maintains photosynthesis and growth of plants under salt stress conditions (12).

### The Sulfur Metabolism Plays a Key Role in Salt Stress Adaptation of Arabidopsis.

**Fig. 5.**
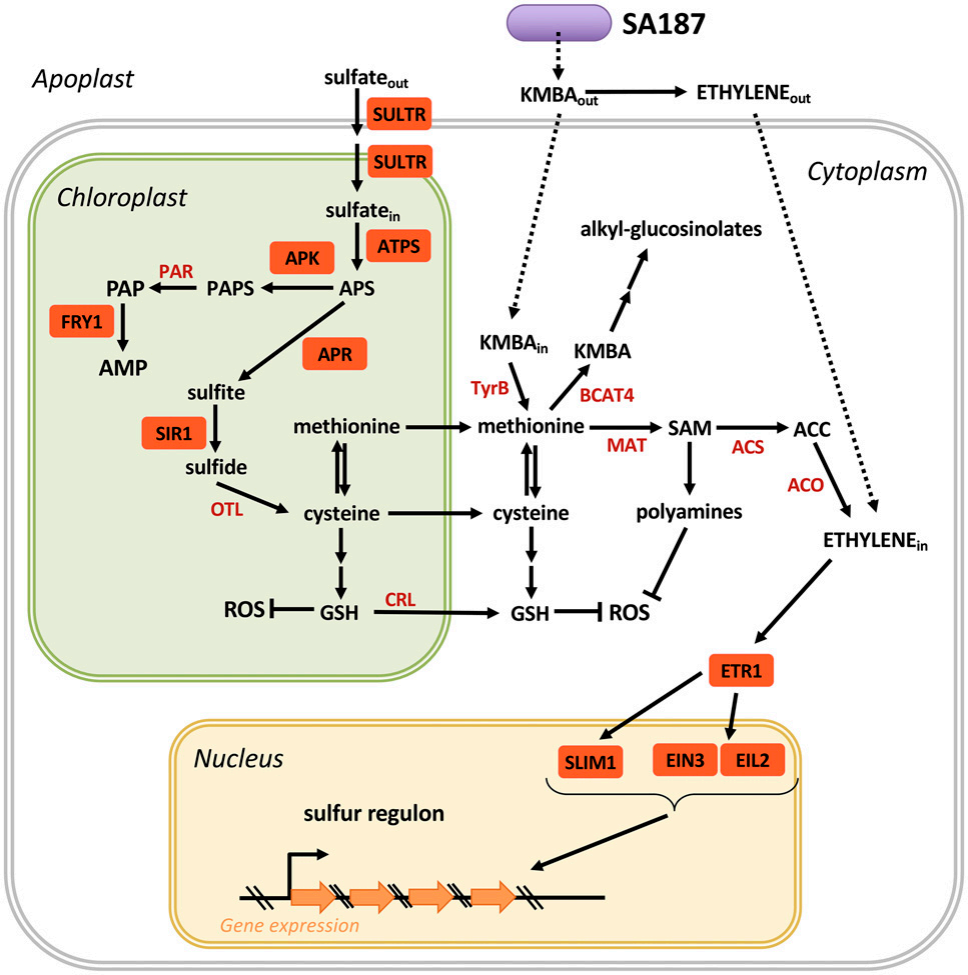
Model of SA187-induced sulfur metabolism and ethylene signaling in Arabidopsis: uptake of sulfate is followed by conversion into APS, sulfite, sulfide, and cysteine, which can be converted into either glutathione (GSH) or methionine. GSH can block salt-induced ROS production in plastids. Methionine can be converted into SAM, ACC, and ethylene, which regulates the sulfur regulon via the transcription factors SLIM1, EIL2, and EIN3. The ethylene-regulated sulfur regulon comprises most genes of the sulfur metabolism, thereby creating a feedback loop between sulfur status and genes encoding sulfur metabolic enzymes. Sulfur status is conveyed by PAP level–induced retrograde signaling to regulate nuclear encoded plastid and sulfur metabolic genes. SULTR = sulfate transporter, ATPS = ATP sulfurylase, APR = APS reductase, APK = APS kinase, PAR = PAPS reductase, SIR1 = sulfite reductase, FRY1 = 5′-bisphosphate, nucleotide phosphatase, OTL = O-acetylserine thiollyase, TyrB = aromatic–amino acid transaminase, BCAT4 = methionine transaminase, MAT = methionine adenosyltransferase, ACS = ACC synthase, ACO = ACC oxidase, ETR1 = ethylene receptor 1, SLIM1 = sulfur limitation1 (central transcriptional regulator of plant sulfur response and metabolism), EIN3 = ethylene-insensitive 3, EIL2 = ethylene-insensitive 3-like 2, CRL = chloroquine-resistance transporter (CRT)-like protein, APS = adenosine 5′-phophosulfate, PAPS = 3′-phosphoadenosine-5′-phosphosulfate, PAP = 3′-polyadenosine 5′-phosphate, AMP = adenosine 5′-monophophfate, KMBA = 2-keto-4-methylthiobutyric acid, SAM = S-adenosyl methionine, ACC = 1-aminocyclopropane-1-carboxylic acid, GSH = glutathione, ROS = reactive oxygen species.

Apart from serving as a building block for the essential amino acids cysteine and methionine, sulfur metabolism is also linked to produce various secondary metabolites. Whereas cysteine serves as a building block for glutathione, methionine is the essential precursor of S-adenosyl-methionine (SAM), which serves as a methyl donor and precursor of a number of processes, including the synthesis of glucosinolates, polyamines, and ethylene (Fig. 5). For this purpose, sulfur uptake and metabolism are tightly controlled. In roots, sulfur is taken up as sulfate that is mainly controlled by the expression of the SULTR transporters. We found that SA187 increases sulfur as well as cysteine and methionine levels in Arabidopsis salt-stressed roots (Fig. 3*A* and *SI Appendix*, S8 *A* and *B*), indicating that SA187 directly affects sulfur uptake and metabolism by Arabidopsis. Once sulfate has been taken up by plant roots, ATPS converts sulfate to APS, which is then first converted to sulfite by APR and sulfide by sulfite reductase (SIR1), before generation of cysteine and methionine in the following reactions. To test the role of the sulfur metabolism and in the beneficial SA187 interaction with Arabidopsis during salt stress, we analyzed *sultr1;2* (34), *apk1 apk2* (35), *apr2* (36), *sir1* (37), and *fry1* (38) mutant plants for their behavior with respect to salt stress and SA187. Although all mutants were little affected in growth under nonsalt conditions (*SI Appendix*, Fig. S8*C*), they showed strong growth inhibition under salt stress (Fig. 3*C*), supporting the notion that the sulfur metabolism plays an essential role in salt stress tolerance. Probably due to redundancy of the sulfur transporters and APR genes, SA187 was able to almost completely rescue the salt growth inhibition phenotype of *sultr1;2* and *apr2* mutants. However, a different result was obtained with mutants of the sulfite reductase SIR1, which is a bottleneck in sulfur metabolism that limits the availability of cysteine, methionine, and their derived downstream products such as glutathione or polyamines (37). SA187 could hardly alleviate the drastic growth inhibition phenotype of *sir1* mutants, indicating that an intact sulfur assimilation pathway is necessary for SA187-induced salt stress tolerance in Arabidopsis.

APS can also be converted to PAPS by several APS kinases, and PAPS serves as a sulfate donor for the enzymatic reactions of numerous sulfotransferases (SOTs) to various substrates, including glucosinolates and phytohormones, thereby generating the side product PAP (Fig. 5). Unstressed plants contain very low levels of PAP mainly due to the activity of FRY1, which dephosphorylates PAP to AMP. However, under stress conditions, PAP levels accumulate and serve as a retrograde signal in coordinating gene expression of nuclear chloroplast genes upon sulfate starvation (42). The central role of FRY1 in linking sulfur metabolism to PAP retrograde signaling inspired us to test whether SA187 mediates salt stress tolerance via the Arabidopsis PAP retrograde signaling pathway. Indeed, as reported before, *fry1* mutant plants were hypersensitive to salt stress (38). However, SA187 was able to suppress the salt stress phenotype of *fry1* plants to a significant degree, suggesting that the sulfur monitoring FRY1 pathway is also involved in mediating the beneficial effect of SA187 on Arabidopsis during salt stress (Fig. 5).

### Coordinate Regulation of Arabidopsis Sulfur Metabolism and Ethylene Signaling by SA187.

In our previous work, we found an essential role of the SA187-derived methionine metabolite KMBA in mediating ethylene-induced plant salt stress tolerance (12). Plant ethylene signaling is linked to the sulfur metabolism via SAM as a precursor of ethylene. There is evidence that genes related to the sulfur metabolism are also regulated by an alternative ethylene pathway that is composed of the ethylene receptor ETR1 and the transcription factor SLIM1 (43). SLIM1 is part of the family of EIN3 transcription factors and is also called EIL3. Although apparently only SLIM1 homodimers can bind to the promoters of the sulfur regulon, SLIM1/EIL3 can also form homo- and heterodimers with the other members of the EIN3 family, allowing fine-tuning of the sulfur metabolism (44). Recently, Dietzen et al. (45) unraveled an important function of the EIL1 and EIL3 transcription factors in the regulation of the sulfur metabolism under sulfur limiting conditions. Although the comparison of transcriptome analysis of our data with those of Dietzen et al. (45) revealed only a 5% overlap in the DEGs of SA187-colonized roots with those of *eil1 eil3* plants, a 33% overlap was found for SA187-colonized shoots. These results require further investigation but support the model that the EIL transcription factors are involved in mediating some of the beneficial responses of the Arabidopsis sulfur metabolism upon SA187 colonization.

The sulfur regulon contains 27 genes that are all related to the sulfur metabolism and are mostly located in the chloroplast (33). Our transcriptome analysis indicated that SA187 regulates several genes of the sulfur regulon in Arabidopsis, in particular ChaC-like family protein and ATSDI1, which were among the significantly responding genes to sulfate starvation in the metatranscriptomic study by Henríquez-Valencia et al. (33) (Fig. 4*D*). To test the hypothesis that bacterially produced KMBA induces ethylene and thereby regulates the sulfur regulon, we performed transcriptome analysis of KMBA- or ACC-treated Arabidopsis plants under salt stress conditions. Comparison of the sulfur regulon transcript levels of KMBA- and ACC-treated Arabidopsis with SA187-colonized plants (Fig. 4 *A–D* and *SI Appendix*, Fig. S4*A*) supports the model that KMBA-produced SA187 regulates the sulfur metabolism of Arabidopsis via the ethylene signaling pathway (Fig. 5).

The sulfur metabolism is not only linked to protein synthesis and ethylene signaling but is also an essential precursor of glutathione biosynthesis, thereby providing a key factor for ROS detoxification. Salt stress is known to induce ROS accumulation in Arabidopsis and other plants and is considered to be one of the major detrimental factors to plants under these conditions. We therefore tested whether SA187 can protect Arabidopsis from enhanced ROS levels under salt stress. Indeed, SA187 could reduce salt stress–induced ROS levels in Arabidopsis. Interestingly, part of the sulfur regulon are four *LSU* genes, encoding chloroplast targeted proteins that directly interact and activate the Fe-Superoxide dismutase FSD2. Importantly, the hypersensitive salt phenotype of amiR-LSUc mutants could be almost completely suppressed by SA187. These data support the role of LSUs as protein hubs linking sulfur metabolism to salt stress–induced chloroplast dysfunction (40) and provide a further link of SA187 and the Arabidopsis sulfur metabolism during salt stress tolerance.

## Conclusions

Overall, our data suggest that upon endophytic interaction of SA187 with Arabidopsis, a nutrient-rich environment is provided by the host plant favoring bacterial growth and proliferation. In exchange, SA187 protects its host from the negative effects of salt stress conditions through providing the metabolite KMBA, which can be converted to ethylene but also other compounds such as methionine or cysteine. Ethylene signaling in Arabidopsis regulates the sulfur regulon, comprising a large fraction of the sulfur metabolic enzymes, thereby regulating sulfate uptake and the generation of cysteine and methionine that serve not only as precursors for protein synthesis but also for the synthesis of the important antioxidants glutathione or polyamines that can help to detoxify ROS. SA187 thereby protects plants from the negative effects of salt stress and maintains photosynthesis and plant growth. Regulation of the sulfur metabolism does not seem to be unique to SA187. Recently, Cheng et al. (46) have identified the APS reductase gene *cysH* as a key factor for the beneficial interaction of *Pseudomonas fluorescens* with Arabidopsis. They also found that the sulfur metabolism of Arabidopsis plays an important role in *P. fluorescens* ISR and growth. Therefore, the coordination of the bacterial and the host plant sulfur metabolisms seems to be central in protecting plants from abiotic and biotic stress conditions.

## Materials and Methods

### Plant Growth Conditions and Inoculation.

*Arabidopsis thaliana* Col-0 was used as wild type. Mutated lines *sultr1;2* (34), *apk1 apk2* double mutant of SALK_053427 (*apk1-1*) and SALK_093072 (*apk2-1*) (35), *apr2* (36), *sir1* (37), *fry1* (38), and amiR-LSUc (39) were also used. Arabidopsis seedlings were grown as described in Saad et al. (47). Briefly, *Arabidopsis* seeds were surface sterilized 10 min in 70% EtOH + 0.05% sodium dodecyl sulfate on a shaker, washed twice in 96% EtOH, and air dried. Sterilized seeds were sown on half-strength Murashige and Skoog (48) (1/2MS) agar plates (0.9% agar) inoculated with SA187 and stratified for 2 d at 4 °C in darkness. After stratification, seeds were germinated vertically for 5 d at 22 °C and 16/8 h light/dark cycle with photon flux density 150 μmol m^−2^ s^−1^ during the light cycle. Uniformly germinated seedlings with 1-cm root length were then transferred and grown vertically in 1/2MS plates without (nonsalt, 1/2MS) or with 100 mM NaCl (salt stress, 1/2MS + 100 mM NaCl) and grown for another 12 d. Six seedlings were transferred to each plate. To prepare the SA187 inoculum, overnight bacterial cultures in Luria–Bertani (LB) broth (Sigma) were harvested, centrifuged 15 min at 3,000 rpm, washed twice in liquid 1/2MS, and resuspended in 1/2MS to a final optical density (OD_600_) = 0.2 (∼10^7^ colony-forming units [CFU]). Plates containing 50 mL of cooled-down 1/2MS or 1/2MS + 100 mM NaCl were inoculated with 0.1 mL of the bacterial suspension (∼10^7^ CFU) and left to solidify. At the end of the experiment, whole 17-d-old plantlets were collected and immediately submerged in liquid nitrogen, then stored at −80 °C until needed.

### Plant Screening Assays.

The salt stress tolerance assays and different ethylene precursors treatments, ACC (100 nM) and KMBA (100 nM), were done as described in de Zélicourt et al. (12) with some modifications. Briefly, 5-d-old colonized seedlings were transferred onto 1/2MS plates with or without 100 mM NaCl (Sigma). Primary root length was measured every 2 d using ImageJ software after scanning the plates. Lateral root density was evaluated as detectable number of lateral roots under a stereo microscope divided by the primary root length. Fresh weight of shoots and roots was measured 17 d after transfer of seedlings. Dry weight was measured after drying shoot and roots for 2 d at 70 °C.

### Bacterial Growth Conditions.

For preparing bacteria-only samples, *Enterobacter* sp. SA187 was grown overnight in LB broth until it reached exponential growth phase. A volume of this preculture was collected, centrifuged, and pelleted cells were washed twice with 1/2MS and resuspended to a final OD_600_ = 0.2 (∼10^7^ CFU). A total of 500 μL of this bacterial suspension was inoculated into 50 mL of 1/2MS or 1/2MS + 100 mM NaCl, and the bacterial culture was incubated 4 h at 28 °C, in darkness. To evaluate the effect of sugar in the bacterial metabolism, broth was supplemented with 1% sucrose. After incubation, 20 mL bacterial culture was harvested, and the cell pellet (∼1.4 × 10^10^ CFU) was stored at −80 °C until needed.

### Sulfur Quantification.

Sulfur was measured for 21-d-old shoot and root dry samples by digestion method, using 2 mL freshly prepared 1% HNO_3_ (trace metal grade, Fisher Scientific) that was added to the preweighed shoot and root samples. After digestion, 1 mL of samples were transferred to volumetric test tubes and diluted to a final volume of 50 mL with 1% HNO_3_. The concentration of sulfur was determined using ICP-OES (Agilent 5110, Agilent Technologies) with the following conditions: 1.2 KW RF power, 0.7 L/min nebulizer flow, 12.0 L/min plasma flow, 1.0 L/min auxiliary flow, argon gas, 8-mm viewing height, and axial viewing mode.

### RNA Extraction.

To determine gene expression in free-living SA187 (“B,” “SB”), total RNA of SA187 cultured in liquid medium was extracted by using RiboPure Bacteria kit (Ambion) following manufacturer’s instructions with the following modifications. During the cell lysis step, no beads were used, and samples were disrupted using the PowerLyzer 24 homogenizer (Mobio) with the following setup: three cycles 30 s “on,” 30 s “off.” To simultaneously capture bacterial and plant transcripts, high-quality total RNA was isolated from 17-d-old SA187 colonized Arabidopsis seedlings (“PB,” “SPB”), as well as separately shoots and roots (“SP,” “SPB,” “SP+ACC,” “SP+KMBA”), by using the Nucleospin RNA plant kit (Macherey-Nagel) as described in de Zélicourt et al. (12). RNA concentration was assessed by using a Qubit 2.0 Fluorometer and the RNA BR assay kit (Invitrogen), and total RNA integrity was verified by using a 2100 Bioanalyzer and the RNA 6000 Nano assay (Agilent).

### RNA-Seq Library Preparation and Sequencing.

As starting material, 1 µg RNA from bacteria samples and 1 µg RNA from dual samples (plant + bacteria) were used. Total RNA from bacteria-only samples were depleted of ribosomal RNA (rRNA) by using Ribo-Zero Magnetic Kit (Bacteria) (Epicentre/Illumina). For rRNA removal of plant + bacteria samples, 1:1 mixture of Ribo-Zero Magnetic Kit (Bacteria) and Ribo-Zero Magnetic Kit (Plant Leaf) was used. After rRNA depletion, RNA-Seq libraries were prepared by using the TruSeq Stranded Total RNA LT kit (Illumina). Final complementary DNA (cDNA) libraries were sequenced by using HiSeq2500 at the Core Lab Bioscience Platform (King Abdullah University of Science and Technology [KAUST], Saudi Arabia). Three biological replicates of each sample were used.

### RNA-Seq Analysis and Quantification of Differential Gene Expression.

Strand-specific paired-end sequencing of RNA-Seq samples was performed by using Illumina HiSeq2500 with a read length of 101 base pair (bp). Reads were quality controlled by using FASTQC (http://www.bioinformatics.babraham.ac.uk/projects/fastqc/), and those with a FASTQC quality score >30 were considered for further analysis. Removal of low quality sequences and adaptor sequences, as well as additional trimming of 5′- and 3′-ends of bacteria derived reads, was performed by using Trimmomatic (49) applying the following parameters: minimum length of 36 bp, mean Phred quality score (Q) greater than 30, leading and trailing bases removal with base quality below 3, sliding window of 4:15. Clean reads were then mapped to the reference genomes by using TopHat (version 2.0.9) (50). Reads derived from “B” and “SB” samples were mapped to the *Enterobacter* sp. SA187 genome (CP019113) downloaded from the in-house INDIGO data warehouse (51), while reads derived from “PB” and “SPB” samples were mapped to a concatenated sequence built up by both SA187 and Arabidopsis genomes. Arabidopsis TAIR10 genome was downloaded from TAIR (https://www.arabidopsis.org). In the present study, only reads that mapped to the bacterial genome were taken into account from dual samples. Transcript assembly, quantification, and differential expression analysis were performed by using Cufflinks version 2.2.0 (52). To overcome the difference of sequence coverage between cultured bacteria and endophytic samples (*SI Appendix*, Table S1), library size normalization was applied before DEG analysis. Sequencing saturation was analyzed to assess whether the sequencing depth was sufficient to ensure the quality of the RNA-Seq experiment. Saturation curves were generated by using the R package RNA-SeQC (53). The reproducibility of the three biological replicates was assessed by calculating the Pearson’s correlation of normalized gene expression in reads per kilobase per million mapped reads. DEG analysis was performed by averaging FPKM from all three biological replicates for each sample. CummeRbund (version 2.0.0) was used for visualization of DEG (53). To identify DEGs, the threshold of q-value ≤0.05 was used. Additionally, genes were considered to be regulated if 0.5> fold change >2.

### RNA-Seq Analysis and Quantification of Differential Gene Expression of Plant Tissues (Shoots and Roots).

Total RNA was extracted from 21-d-old plant tissues shoots and roots in presence or absence of SA187, treated with KMBA or ACC under 100 mM NaCl using Nucleospin RNA plant kit (Macherey-Nagel) as described in de Zélicourt et al. (12). RNA samples were sequenced using Illumina deep sequencing. Three biological replicates were sequenced per condition. Samples were sequenced using HiSeq4000 with a read length of 150 bps. Sequenced reads were checked for quality using FASTQC (54). Trimmomatic version 0.36 (49) was used to filter low quality reads and bps with the following parameters: Minimum length of 36 bp; Mean Phred quality score greater than 30; Leading and trailing bases removal with base quality below 3; Sliding window of 4:15. Trimmed reads were aligned to TAIR10 (55) genome using TopHat version 2.0.9 (56). Aligned reads were then used to calculate the number of reads per gene using FeatureCounts version 1.6.5 (57), and FPKM was calculated using Cufflinks version 2.2.1 (58). The differential expression between two samples was calculated using Cuffdiff from Cufflinks version 2.2.1 (58) package. To identify the DEGs, specific parameters (q-value 0.05 and statistical correction: Benjamini Hochberg) were used. Genes that show 0.5> fold change >2 and q-value ≤0.05 to the mock were considered to be regulated. Regulated genes functional annotation was carried out using AgriGO2 (59). Common genes that are up-/down-regulated between different comparisons are done using Venny2 (60).

### RNA-Seq Validation by qRT-PCR.

To validate the results derived from the bacterial single and dual RNA-Seq experiments, 50 DEGs were selected and their gene expression analyzed by qRT-PCR by using specific primers (*SI Appendix*, Table S2). Primers were designed by using Primer-Blast on-line tool available at the National Center for Biotechnology Information (NCBI) (https://www.ncbi.nlm.nih.gov/tools/primer-blast/). The translation initiation factor IF-2 (*infB*) was used as reference gene (12). The cDNAs used as template for qRT-PCR were synthesized by using SuperScriptIII (Invitrogen) and oligo-dT following manufacturer’s recommendations. qRT-PCR reactions were carried out in a CFX96 Touch Real-Time PCR Detection System (BIO-RAD) as follows: 95 °C for 10 min; 40 × (95 °C for 10 s and 60 °C for 40 s) followed by a dissociation step (melting curve) to validate the PCR products. Reactions were performed in three technical replicates from each biological replicate, and the relative gene expression was calculated using the ΔΔC_T_ method.

For the qRT-PCR analysis of plant tissues roots and shoots, cDNAs were synthesized by using SuperScriptIII (Invitrogen) and oligo-dT following manufacturer’s recommendations. qRT-PCR reactions were carried out in a CFX384 Real-Time PCR Detection System (BIO-RAD) as follows: 50 °C for 2 min; 95 °C for 10 min; 39× (95 °C for 10 s and 60 °C for 40 s) followed by a dissociation step (melting curve) to validate the PCR products. Reactions were performed in three technical replicates from each biological replicate. The reference genes used in this analysis were UBIQUITIN10 (At4g05320) and ACTIN2 (At3g18780) (33), and the relative gene expression was calculated using the Bio-Rad CFX manager software.

## Data Availability

RNA-Seq data are available at NCBI/Gene Expression Omnibus (GEO) DataSets (https://www.ncbi.nlm.nih.gov/gds) under the accession nos. GSE124591 (free-living SA187), GSE102950 (Arabidopsis-associated SA187) (12), GSE133175 (Arabidopsis tissues: shoot/root associated SA187), and GSE145884 (Arabidopsis tissues: shoot/root treated with ACC or KMBA). All other study data are included in the article and/or supporting information.
